# Phylogenetic analysis of forkhead transcription factors in the Panarthropoda

**DOI:** 10.1007/s00427-022-00686-3

**Published:** 2022-03-01

**Authors:** Christoph Schomburg, Ralf Janssen, Nikola-Michael Prpic

**Affiliations:** 1 Fachgebiet Botanik, Institut Für Biologie, Universität Kassel, Heinrich-Plett-Straße 40, 34132 Kassel, Germany; 2 Institut Für Allgemeine Zoologie Und Entwicklungsbiologie, AG Zoologie Mit Dem Schwerpunkt Molekulare Entwicklungsbiologie, Justus-Liebig-Universität Gießen, Heinrich-Buff-Ring 38, 35392 Gießen, Germany; 3 Department of Earth Sciences, Uppsala University, Villavägen 16, 75236 Uppsala, Sweden

**Keywords:** Fox genes, Phylogeny, Gene duplication, Forkhead domain, Panarthropods

## Abstract

Fox genes encode transcription factors that contain a DNA binding domain, the forkhead domain, and are known from diverse animal species. The exact homology of the Fox genes of different species is debated and this makes inferences about the evolution of the Fox genes, and their duplications and losses difficult. We have performed phylogenetic analyses of the Fox gene complements of 32 panarthropod species. Our results confirm an ancestral complement of FoxA, FoxB, FoxC, FoxD, FoxF, FoxG, FoxJ1, FoxJ2/3, FoxK, FoxL1, FoxL2, FoxN1/4, FoxN2/3, FoxO, FoxP, and FoxQ2 in the Arthropoda, and additionally FoxH and FoxQ1 in the Panarthropoda (including tardigrades and onychophorans). We identify a novel Fox gene sub-family, that we designate as FoxT that includes two genes in *Drosophila melanogaster*, *Circadianly Regulated Gene* (*Crg-1*) and *forkhead domain 3F* (*fd3F*). In a very recent paper, the same new Fox gene sub-family was identified in insects (Lin et al. 2021). Our analysis confirms the presence of FoxT and shows that its members are present throughout Panarthropoda. We show that the hitherto unclassified gene *CG32006* from the fly *Drosophila melanogaster* belongs to FoxJ1. We also detect gene losses: FoxE and FoxM were lost already in the panarthropod ancestor, whereas the loss of FoxH occurred in the arthropod ancestor. Finally, we find an ortholog of FoxQ1 in the bark scorpion *Centruroides sculpturatus*, confirmed not only by phylogenetic analysis, but also by forming an evolutionarily conserved gene cluster with FoxF, FoxC, and FoxL1. This suggests that FoxQ1 belongs to the ancestral Fox gene complement in panarthropods and also in chelicerates, but has been lost at the base of the mandibulate arthropods.

**Supplementary Information:**

The online version contains supplementary material available at 10.1007/s00427-022-00686-3.

## Introduction

Phenotypic diversity of all organisms is achieved through changes in developmental genetic programs. These processes are governed by genetic networks, which usually have transcription factors at the nodes of these networks. For new genetic networks to arise, existing genes are either co-opted from other networks, or new functions are introduced by the expansion of existing gene families through duplication and subsequent neo-functionalization (Lynch and Conery 2000). One family of genes that have been expanded in particular are the forkhead box genes (Fox genes). They are present with at least one family member found in opisthokont lineages such as Ichthyosporea (Suga et al. 2013), but have more than 40 members in mammals (e.g., Katoh and Katoh 2004).

The first forkhead domain gene to be identified was *fork head* itself in the fly *Drosophila melanogaster* (Weigel et al. 1989), followed shortly by one of its homologs in the rat (Lai et al. 1990). Both genes were found to code for a similar helix-turn-helix motif DNA binding domain, which was termed winged-helix (Li and Tucker 1993). Subsequently, it could be shown that this 110 amino acid forkhead domain is widely conserved among different taxa (Kaufmann and Knöchel 1996) and that genes containing this domain are widespread throughout the animal kingdom. The identified forkhead genes have been shown to fulfill diverse roles during embryonic development, cell fate decisions, morphogenesis, cell cycle control, metabolism, signal transduction, or the change of chromatin state (e.g., Carlsson and Mahlapuu 2002; Pohl and Knöchel 2005).

With the identification of more and more forkhead genes, it became apparent that many of these independently discovered genes actually form groups of homologs shared between deuterostomes, lophotrochozoans, ecdysozoans, and non-bilaterian metazoans (Larroux et al. 2008; Magie et al. 2005; Mazet et al. 2003). This discovery led to the introduction of a unified nomenclature for forkhead gene sub-families using the letters of the Latin alphabet to denote homologous genes in diverse organisms. Initially, sub-families were named from FoxA to FoxO (Kaestner et al. 2000), but new sub-families were subsequently added up to FoxT, some subgroups have been further subdivided (e.g., FoxQ1 and FoxQ2), and some Fox genes are difficult to place into a sub-family (e.g., *fd3F*). Therefore, the exact number of Fox families is still debated, ranging from 17 to well over 20 (Larroux et al. 2008; Lin et al. 2021; Mazet et al. 2003; Hannenhalli and Kaestner 2009; Shimeld et al. 2010a, b; Tu et al. 2006). Newly identified forkhead domain genes were usually compared to the Fox repertoire of well-studied model organisms, such as the fly *Drosophila melanogaster* or the mouse *Mus musculus*. Thus, many genes which were not found to be homologs of any known Fox family were assigned an “orphan state,” for instance in the nematode *Caenorhabditis elegans* (Hope et al. 2003), the hemichordate *Saccoglossus kowalewskii* (Fritzenwanker et al. 2014), and several lophotrochozoans (e.g., Yang et al. 2014). Other approaches focused on the Fox gene repertoire in a single lineage and classified forkhead homologs only in *Drosophila melanogaster* (Lee and Frasch 2004), or among vertebrates (Kaestner et al. 2000). Several studies have tried to unravel the phylogenetic history of forkhead genes and link their emergence to the introduction of evolutionary novelties (e.g., Larroux et al. 2008; Mazet et al. 2003; Shimeld et al. 2010b). These studies are all dependent on the correct initial identification of forkhead genes and therefore these studies potentially overlooked orphan genes, which might belong to a previously unidentified new family or derived members of an existing family, due to the lack of comparable sequences. With the ever-increasing availability of sequenced genomes and embryonic transcriptomes from a wide variety of species, it is now possible to compare the Fox gene repertoire of many different taxa at the same time.

The existence of clustered genes in a genome has been reported for several gene families, the most prominent example being the Hox cluster (e.g., Garcia-Fernàndez 2005; Lemons and McGinnis 2006). Clusters of genes of the same family are thought to arise via tandem duplication from one ancestral gene (e.g., Shimeld et al. 2010b). In the case of Fox genes, a close association has been observed for the two paralogs of *sloppy-paired* in *Drosophila melanogaster* (Cadigan et al. 1994). In addition, the genes FoxQ1, FoxF, FoxC, and FoxL1 form a cluster in the genomes of many different lineages (Shimeld et al. 2010a; Mazet et al. 2006; Wotton et al. 2008; Yu et al. 2008a, b). This suggested that this cluster had evolved by tandem duplication of an ancestral gene and was already present in the bilaterian ancestor (Mazet et al. 2006). Traces of this cluster can be found throughout the Bilateria, but the reason for its maintenance remains unknown. It has been proposed that the co-linearity of genomic arrangement and expression patterns may act as a selective force for the maintenance of such a cluster, as seen for the Hox genes (Monteiro and Ferrier 2006), or that their co-expression in the same tissue might explain their presence at the same chromosomal location, where they form a regulatory block of chromatin (Shimeld et al. 2010a).

Our aim was to identify members of the forkhead family in members of panarthropods (Arthropoda, Tardigrada, and Onychophora), to assign them to their respective sub-families by phylogenetic sequence analysis and to analyze their chromosomal location (if known) and identify possible clusters of Fox genes in order to gain insights into the evolutionary history of this gene family.

## Results and discussion

### Fox gene complements in diverse panarthropods

We have searched the genomic or transcriptomic sequence resources of 32 panarthropod species including representatives of the Tardigrada and Onychophora (see list of species in Table 1) for the presence of genes that contain a forkhead domain. The number of Fox genes identified in the genome/transcriptome sequences varied between 10 genes and a maximum of 50 genes. Usually, panarthropods have at least 16 Fox genes (Fig. 2, rightmost column). However, a few species have a smaller complement of Fox genes. Only 10 Fox genes have been found in the locust *Locusta migratoria*, 12 Fox genes have been found in the woodlouse *Armadillidium vulgare*, and 14 Fox genes have been found in the tardigrade *Ramazzottius varieornatus*, the dipluran *Catajapyx aquilonaris*, the springtail *Folsomia candida*, and the honey bee *Apis mellifera*. It is difficult to judge whether these low numbers represent a genuinely reduced Fox gene complement in these species, or are an artifact caused by incomplete genome assembly in these species. At least in *Locusta migratoria*, the unusually low Fox gene number suggests the latter, whereas the tardigrade genome has been shown previously to have undergone strong reduction including significant gene loss (Bemm et al. 2017; Yoshida et al. 2017; Guijarro-Clarke et al. 2020), thus making the evolutionary loss of Fox genes likely.

**Table 1:** List of panarthropod species used in the analysis. A total of 32 species was used, and for each species, the scientific name and a common name are given

Tardigrades	Apterygote hexapods
*• Ramazzottius varieornatus* (water bear)	*• Folsomia candida* (white springtail) *• Catajapyx aquilonaris* (forcepstail)
Onychophorans	Basal pterygote insects
*• Euperipatoides kanangrensis* (velvet worm)	*• Ephemera danica* (mayfly) *• Ladona* (or *Libellula*) *fulva* (dragonfly)
Chelicerates	Hemimetabolous insects
*• Limulus polyphemus* (horseshoe crab) *• Centruroides sculpturatus* (bark scorpion) *• Parasteatoda tepidariorum* (cobweb spider) *• Varroa destructor* (bee mite) *• Tetranychus urticae* (spider mite)	*• Medauroidea extradentata* (stick insect) *• Blatella germanica* (cockroach) *• Locusta migratoria* (migratory locust) *• Pediculus humanus* (body louse) *• Frankliniella occidentalis* (flower thrips) *• Laodelphax striatellus* (brown planthopper) *• Halyomorpha halys* (marmorated shield bug) *• Rhopalosiphum maidis* (corn aphid)
Myriapods	
*• Strigamia maritima* (coastal centipede) *• Glomeris marginata* (pill millipede)	
Crustaceans	Holometabolous insects
*• Eurytemora affinis* (copepod) *• Daphnia magna* (water flea) *• Penaeus* (or *Litopenaeus*) *vannamei* (king prawn) *• Hyalella azteca* (amphipod) *• Armadillidium vulgare* (woodlouse)	*• Apis mellifera* (honeybee) *• Tribolium castaneum* (flour beetle) *• Limnephilus lunatus* (caddisfly) *• Papilio xuthus* (swallowtail butterfly) *• Ctenocephalides felis* (cat flea) *• Drosophila melanogaster* (vinegar fly)

By contrast, a few species show a very high number of Fox genes. The scorpion *Centruroides sculpturatus* and the cat flea *Ctenocephalides felis* have more than 30 Fox genes, and the horseshoe crab *Limulus polyphemus* has the maximum number of all studied panarthropod species with 50 Fox genes. In the scorpion and the horseshoe crab, this increase in Fox genes is likely to be linked to genome duplications that have been detected in these animal groups (e.g., Kenny et al. 2016; Schwager et al. 2017; Harper et al. 2020). The increase of Fox genes in the cat flea may instead be caused by extensive tandem duplications of single Fox genes (Driscoll et al. 2020).

### Phylogeny of panarthropod Fox genes

**Fig. 1:**
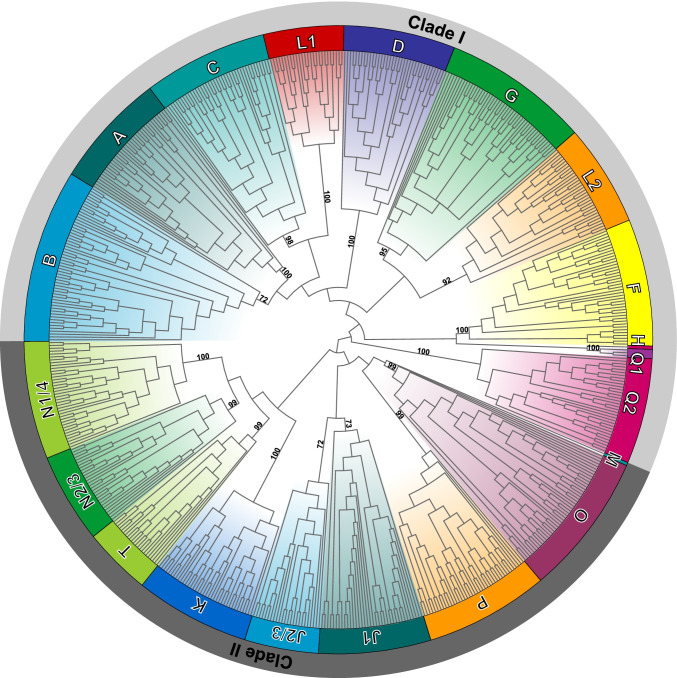
Unrooted phylogenetic cladogram of all Fox genes from representatives of the Panarthropoda, based on the entire sequence of the conceptually translated proteins. The colors denote the Fox-gene sub-families. “Clade I” and “Clade II” indicate the principal subdivision of Fox genes into a group with intronless forkhead domain (Clade I) and a group with the forkhead domain interrupted by at least one intron (Clade II). Species and sequence accession numbers are omitted for lack of space, but are included in Supplementary Figure S3. Numbers at the tree edges indicate summarized bootstrap values according to the Majority Rule

**Fig. 2:**
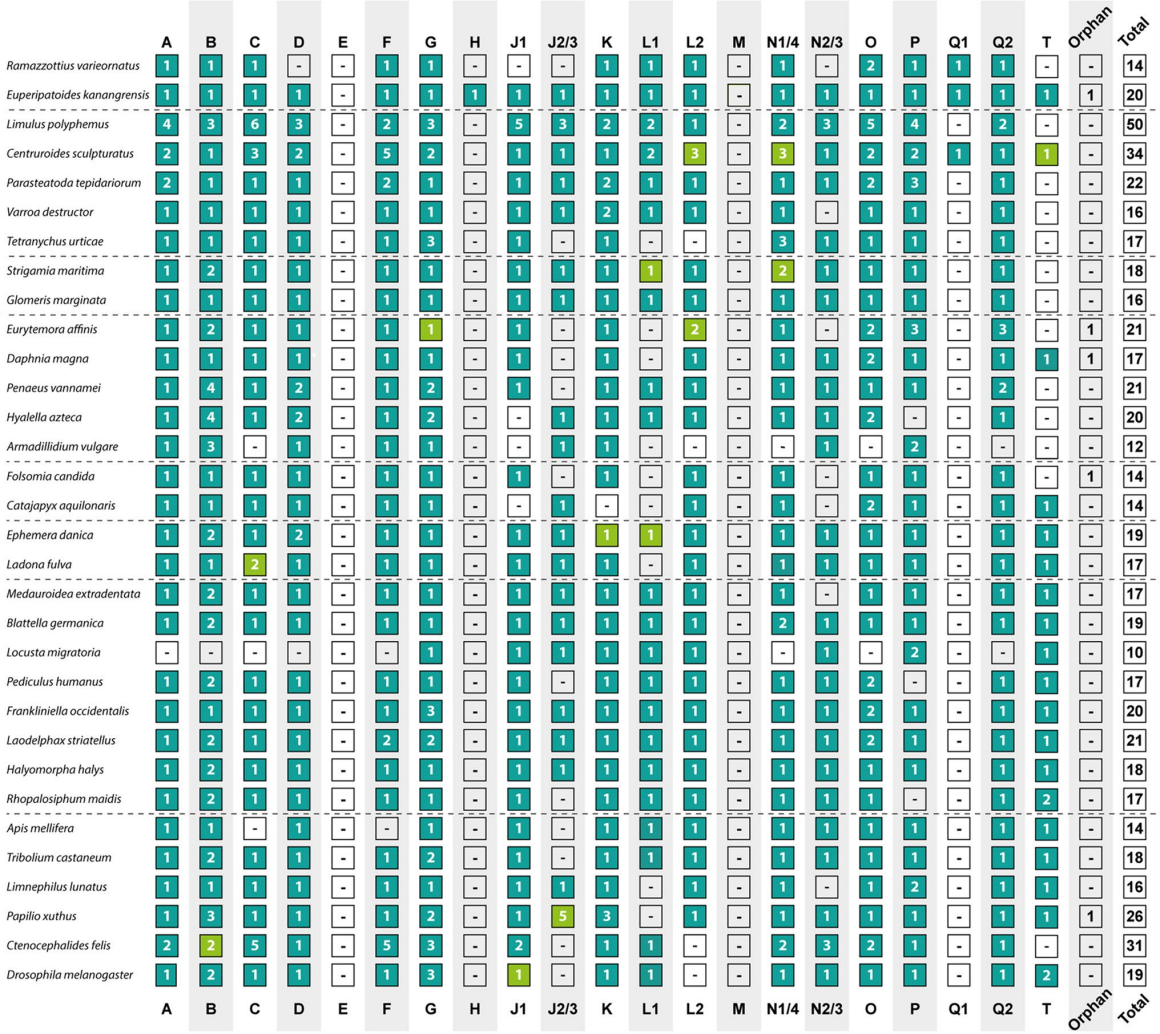
Summary of the Fox gene complement of the panarthropod species used in the analysis. The top row gives the name of the Fox gene sub-family; the numbers in the boxes give the count of duplicated genes in a given sub-family per species; a dash (-) indicates that no clear-cut homologs of this particular sub-family could be identified in the genome of the respective species. The panarthropod Fox gene sequences were included in three separate phylogenetic analyses (see text and Fig. S1 to Fig. S3). Dark green boxes indicate that the respective genes were assigned to this sub-family in all three of these analyses. Light green boxes indicate that the respective genes were assigned to this sub-family in two out of the three analyses. All remaining genes that could not be confidently placed in one of the sub-families in the three phylogenetic analyses are given in the column “Orphan” and the total number of Fox genes per species is given in the column “Total”

We then used the Fox gene sequences from the 32 panarthropod species supplemented with selected additional species from diverse groups of the Opisthokonta (see Supplementary Table S1 for a list of all selected species, and see Supplementary Table S2 for a list of all Fox genes used in the analyses) for phylogenetic reconstruction in order to be able to place the panarthropod Fox sequences into the established Fox gene sub-families. We have reconstructed the phylogeny of all Fox genes from all of the selected taxa based on the sequence of their forkhead domains (Fig. S1), and we have also reconstructed the phylogeny of all Fox genes from the selected panarthropods only, both based on the sequence of the forkhead domain (Fig. S2), and based on the entire gene sequences (Fig. 1, Fig. S3). The detailed results of Fox gene sub-family assignments of all panarthropod Fox gene sequences are shown in Table S3 and a summary overview is shown in Fig. 2. In most cases, the assignment of a Fox gene to a certain sub-family was consistent across all three phylogenetic analyses; these results are shown with the dark green background color in Fig. 2. In some cases, only two of the three phylogenetic analyses agreed on the sub-family assignment; these cases are indicated by the light green background color in Fig. 2. Finally, in some cases the assignment to sub-family differed in all three analyses; these Fox gene sequences could therefore not be assigned to a sub-family and are included in the column “orphan” in Fig. 2.

Based on the results of the phylogenetic sequence analysis, the ancestral complement of arthropod Fox genes includes at least one member of FoxA, FoxB, FoxC, FoxD, FoxF, FoxG, FoxJ1, FoxJ2/3, FoxK, FoxL1, FoxL2, FoxN1/4, FoxN2/3, FoxO, FoxP, and FoxQ2. In addition, there is one further monophyletic group that only comprises panarthropod Fox gene sequences. In the sequence analysis, together with non-panarthropod opisthokonts, this monophyletic group is nested within the FoxN1/4 clade, but in the panarthropod-only analyses this group forms a well-separated monophylum, related to but clearly distinct from FoxN1/4. This new subgroup of Fox genes was also very recently identified by another research group, and was named FoxT, in accordance with the recommendations for a unified nomenclature of Fox genes (Lin et al. 2021). Our new data now reveal that FoxT is not insect-specific (Lin et al. 2021), but that it has members in other arthropods and even an onychophoran species (summarized in Fig. 2). This means that FoxT was present in the last common ancestor of Panarthropoda, but was apparently lost independently in several lineages of arthropods. In most lineages of insects, however, FoxT has been retained, suggesting an important and conserved function of this Fox gene in insects. The function of FoxT is currently only known for the *Drosophila melanogaster* homolog of the FoxT sub-family, *fd3F* (see also below), which is required for the development of the mechanosensory cilium of chordotonal neurons (Newton et al. 2012). The development of mechanosensory cilia, however, is not a panarthropod-specific process, and therefore the origin of FoxT may correlate with a previously unidentified common role in the biology of Panarthropoda. Interestingly, FoxT appears to be a male-specific gene in the brown plant hopper *Nilaparvata lugens* where it is predominantly (if not exclusively) expressed in male nymphs and adult males (Lin et al. 2021). It is thus likely that FoxT is involved in motile cilia development of sperm.

The Fox genes FoxI, FoxR, and FoxS have evolved in the deuterostome/vertebrate lineage (Tu et al. 2006; Wotton et al. 2008; Yu et al. 2008a; b) and are therefore not present in panarthropods, as expected. However, FoxE, FoxH, FoxM, and FoxQ1 are more widely distributed in the metazoans (see Fig. S1) and therefore their lack in (most) arthropods/panarthropods indicates specific events of gene loss. FoxE has apparently been lost already in the panarthropod ancestor, because FoxE is also absent in the onychophoran *Euperipatoides kanangrensis* and the tardigrade *Ramazzottius varieornatus*. FoxH is absent from arthropods, but this seems to be an arthropod-specific event of gene loss, because a clear ortholog of FoxH is present in *Euperipatoides kanangrensis*.

Another Fox gene absent from arthropods is FoxM, and this Fox gene is apparently also absent from tardigrades and onychophorans. However, there is one Fox gene in *Euperipatoides kanangrensis*, namely *c200914_g1_i1*, that we could not confidently place in a sub-family, and this gene is actually included in FoxM in the phylogenetic analysis that also includes non-panarthropods. In the panarthropod-only analyses, *c200914_g1_i1* is included with FoxJ2/3 when only the forkhead domain is used, but it is placed at the base of FoxO when the entire sequence is used. The latter placement also indicates that *c200914_g1_i1* could actually be a representative of FoxM, because genuine FoxM genes are closely related to FoxO and FoxP (see Fig. S1). More information about the Fox gene complement of additional onychophorans is required to establish whether FoxM is present in non-arthropod panarthropods.

Finally, an intriguing case of gene loss is represented by FoxQ1. This gene is absent from all investigated arthropods, except for the bark scorpion *Centruroides sculpturatus*. The identity of the scorpion gene as a genuine member of FoxQ1 is confirmed not only by the phylogenetic sequence analysis, but also by the clustering of this gene with FoxF, FoxC, and FoxL1 (see below). Thus, at least in the chelicerates, the presence of FoxQ1 appears to represent the ancestral state, and FoxQ1 is also present in the basally branching panarthropods. The loss of FoxQ1 therefore appears to be specific for the myriapods, crustaceans, and insects and might represent an apomorphy for the Mandibulata.

### Fox genes in the arthropod model species *Drosophila melanogaster*

The Fox gene complement of the main arthropod model system, the fly *Drosophila melanogaster*, has been analyzed previously (Lee and Frasch 2004), and FlyBase lists 19 genes with a Fork head domain (Thurmond et al. 2019). Most of these genes are readily assigned to one of the Fox gene sub-families, but some genes proved to be difficult to classify in previous studies (Mazet et al. 2003; Lee and Frasch, 2004). In our analysis, we were able to assign all 19 Fox genes of *Drosophila melanogaster* to sub-family. The assignment of most of the Fox genes is non-controversial and is consistent with previous analyses: *fork head* (*fkh*) is assigned to FoxA*, fd96Ca* and *fd96Cb* are assigned to FoxB, *crocodile* (*croc*) is assigned to FoxC, *fd59A* is assigned to FoxD, *biniou* (*bin*) is assigned to FoxF, and *sloppy paired 1* (*slp1*) and *sloppy paired 2* (*slp2*) are assigned to FoxG. The gene *fd19B* was previously unassigned to a sub-family, but previous analyses have already demonstrated a close relationship of *fd19B* with the two *slp* genes (e.g., Lee and Frasch 2004; Pascual-Carreras et al. 2021), and our analysis confirms this assignment of *fd19B* to FoxG. The assignment of the *Drosophila melanogaster* genes *FoxK*, *FoxL1*, *foxo*, and *FoxP* to the sub-families of the same name was also confirmed in our analysis. In addition, the assignment of *jumeau* (*jumu*) and *Checkpoint suppressor 1-like* (*CHES-1-like*) to FoxN1/4 and FoxN2/3, respectively, was also confirmed. The gene *fd102C* was previously tentatively assigned to FoxQ2 (Lee and Frasch 2004) and we confirm this assignment in our present analysis. The assignment of the gene *CG32006* was previously unclear; it was not assigned to a sub-family by previous studies (e.g., Mazet et al. 2003; Vij et al. 2012), or was assigned to FoxM (Pascual-Carreras et al. 2021). In our analysis, it is firmly placed in the FoxJ1 sub-family, which is notable for its highly conserved role in the formation of motile cilia (Vij et al. 2012; Stubbs et al. 2008; Yu et al. 2008a; b). Our finding of a FoxJ1 homolog in *Drosophila melanogaster* is therefore surprising, because *Drosophila melanogaster* lacks motile cilia in all somatic cells, except for bipolar neurons, and was previously believed to have no FoxJ1 homolog (Vij et al. 2012). However, although our phylogenetic analysis suggests that *CG32006* is a homolog of FoxJ1, the sequence of *CG32006* is also rather diverged from the other members of this Fox sub-family, indicating that *CG32006* might have lost its conserved role in motile cilia formation in *Drosophila melanogaster*. Interestingly, another Fox gene with previously unclear assignment, *fd3F*, appears to take over the role of FoxJ1 in the bipolar chordotonal neurons (Newton et al. 2012). The *fd3F* gene, however, does not group with the FoxJ1 sub-family (as would be expected from its function in motile cilia formation), but is a representative of the newly recognized Fox gene sub-family, FoxT (Lin et al. 2021), that is present throughout the Panarthropoda, and in *Drosophila melanogaster* includes a second previously unassigned Fox gene, *Circadianly Regulated Gene* (*Crg-1*) (Lin et al. 2021).

### Clustering of Fox genes in the genome

**Fig. 3:**
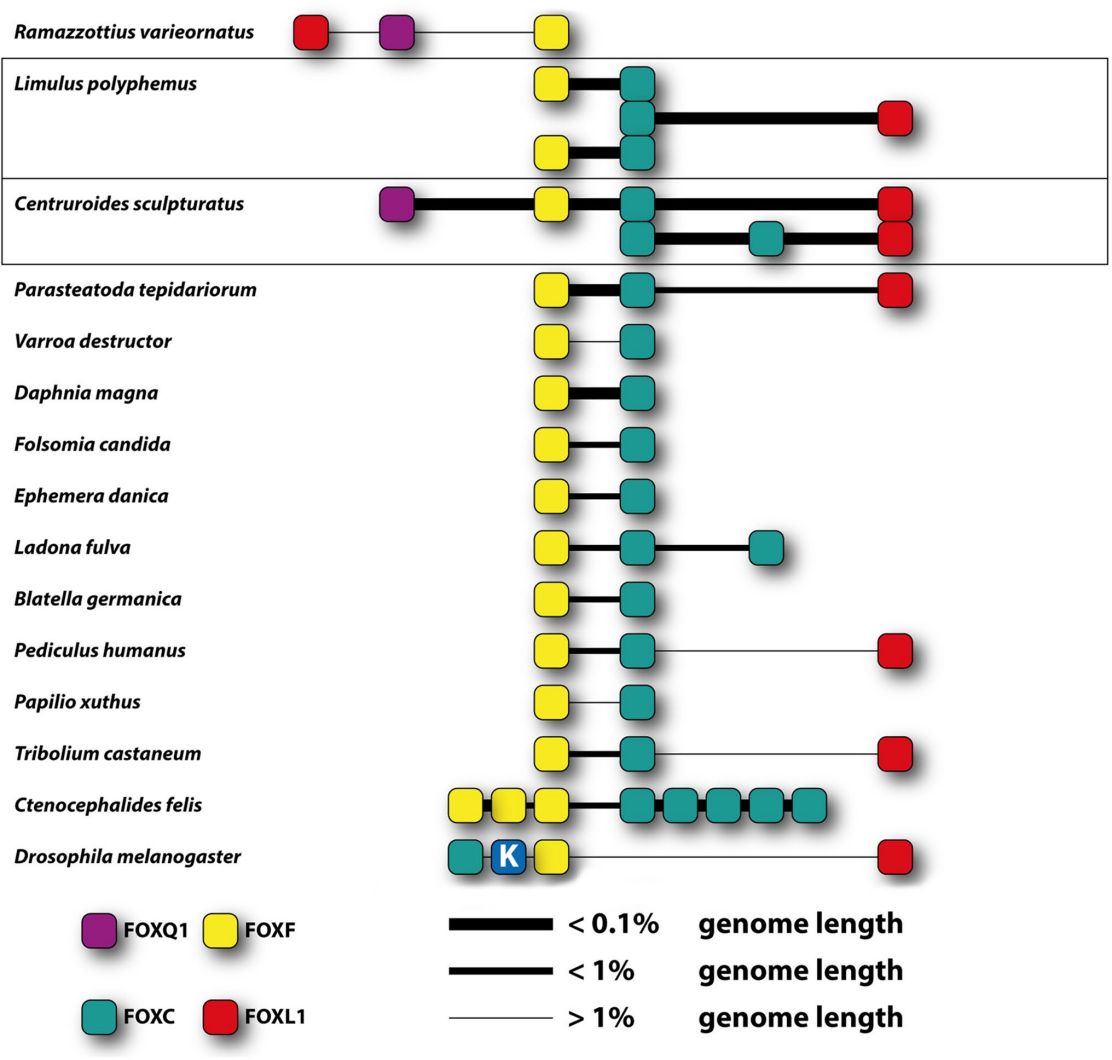
Summary of the chromosomal clustering of the Fox genes FoxQ1 (purple), FoxF (yellow), FoxC (green), and FoxL1 (red) in the genome of panarthropods. The distance (relative to the genome size) between the genes is indicated by the width of the line connecting the genes, with thick lines indicating close proximity (see legend in the figure). Boxes denote duplicated clusters within one species. Note that the cluster in *Drosophila melanogaster* also contains FoxK. See Fig. S4 for details about accession numbers and chromosomal distances

We have also analyzed the location of the Fox genes in the genomes of those panarthropod species for which sufficient linkage information is available. It has been reported previously that the genes FoxQ1, FoxF, FoxC, and FoxL1 are often clustered together in the genome (Shimeld et al. 2010a, b; Mazet et al. 2006). We find partial conservation of this cluster in many panarthropod species (Fig. 3, Supplementary Fig. S4). Interestingly, we find a complete Q1-F–C-L1 cluster in the scorpion *Centruroides sculpturatus*, and an almost complete cluster (lacking only FoxC) in the tardigrade *Ramazzottius varieornatus*, indicating that this cluster was present in the last common ancestor of all arthropod groups and also in the panarthropod ancestor. In insects and crustaceans, FoxF and FoxC are always clustered and FoxL1 is still within the cluster in some species, e.g., the louse *Pediculus humanus*, the beetle *Tribolium castaneum*, and the fly *Drosophila melanogaster*, but FoxQ1 has been lost entirely not only from this cluster, but also from the genome.

## Methods

### Selected species and genome/transcriptome sequence resources

For this analysis, we selected high-quality genomes from species at important systematic positions. We used genomes and their translated protein sequences (official gene sets) from publicly available resources (Poelchau et al. 2015; Evans et al. 2013; Agarwala et al. 2018; Howe et al. 2020), except for the two transcriptomes of the onychophoran *Euperipatoides kanangrensis* (Janssen and Budd 2013) and the millipede *Glomeris marginata* (Janssen and Posnien 2014) (see Supplementary Table S1 for details on sources and Supplementary Table S2 for accession numbers).

Identification of forkhead domain–containing genes.

To identify forkhead domain–containing genes for further analysis, we used the hmmscan function of the HMMER3 software (version 2.3.1) (Eddy 2011) package to scan the downloaded gene sets against the PfamA database (release Pfam30.0) (El-Gebali et al. 2019) for conserved forkhead domains. We set a score of 20 as a cut-off for inclusion in the further analysis, since the lowest scoring forkhead gene, which was previously published, is *FoxR1* from the mouse *Mus musculus* with a score of 23.4. We identified isoforms of genes via the corresponding gff annotation files and chose the sequence with the highest forkhead domain score at each genomic locus. In case of multiple sequences on the different scaffolds/chromosomes of a species, we noted their respective positions and distances relative to one another, in order to see clustered forkhead genes. Information on accession numbers, genomic location, distances, and forkhead domain scores of the selected sequences are listed in Supplementary Table S2.

### Sequence alignment and phylogenetic analysis

From the identified genes, we extracted the forkhead domains as predicted by hmmscan and used the sequences of the domains and the full-length protein sequences for the alignments, using ClustalOmega (Sievers and Higgins 2014) with standard settings. We inferred phylogenetic trees on the alignments using RAxML (version 8.2.11) (Stamatakis 2014; Stamatakis 2015). Since the forkhead domain genes show an extremely conserved domain, and the mapping of bootstrap trees on a maximum-likelihood tree resulted in very low support values (not shown), we decided to summarize the bootstrap trees (-b, 1,000 trees each) with the Majority Rule Consensus function (Wilkinson 1996; Stamatakis and Izquierdo-Carrasco 2011) of RAxML (-J MR). The alignment of the forkhead domain of all arthropod, onychophoran, and tardigrade sequences used in the analysis is provided in Supplementary Figure S5.

## Supplementary Information

Below is the link to the electronic supplementary material.

Supplementary file1 (PDF 35.6 MB) Supplementary Figure S1. Unrooted phylogenetic cladogram of all Fox genes from all selected opisthokont species, based on the sequence of the forkhead domain. The colors denote the Fox-gene sub-families. "Clade I" and "Clade II" indicate the principal subdivision of Fox genes into a group with intronless forkhead domain (Clade I) and a group with the forkhead domain interrupted by at least one intron (Clade II). Species and sequence accession numbers are indicated at the terminals. Numbers at the tree edges indicate summarized bootstrap values according to the Majority Rule. The stars at the tip of the lineages denote sequences derived from arthropods, onychophorans and tardigrades.

Supplementary file2 (PDF 898 KB) Supplementary Figure S2. Unrooted phylogenetic cladogram of all Fox genes from representatives of the Panarthropoda, based on the sequence of the forkhead domain. The colors denote the Fox-gene sub-families. "Clade I" and "Clade II" indicate the principal subdivision of Fox genes into a group with intronless forkhead domain (Clade I) and a group with the forkhead domain interrupted by at least one intron (Clade II). Species and sequence accession numbers are indicated at the terminals. Numbers at the tree edges indicate summarized bootstrap values according to the Majority Rule.

Supplementary file3 (PDF 906 KB) Supplementary Figure S3. Unrooted phylogenetic cladogram of all Fox genes from representatives of the Panarthropoda, based on the entire sequence of the conceptually translated proteins. The colors denote the Fox-gene sub-families. "Clade I" and "Clade II" indicate the principal subdivision of Fox genes into a group with intronless forkhead domain (Clade I) and a group with the forkhead domain interrupted by at least one intron (Clade II). Species and sequence accession numbers are indicated at the terminals. Numbers at the tree edges indicate summarized bootstrap values according to the Majority Rule.

Supplementary file4 (JPG 1151 KB) Supplementary Figure S4. Summary of the FoxQ1-FoxF-FoxC-FoxL1 cluster identified in the available genome sequence of diverse panarthropods. Dashed or full lines separate species. Genome sequence accessions (or chromosomes) are given in the second column, whereas accession numbers for single genes are given below the colored boxes for each gene separately. The numbers above the lines between colored boxes give the distance between the genes (in base pairs). The width of the lines indicates the relative distance: thick lines, less than 0.1% genome length apart; medium lines, less than 1% genome length apart; thin lines, over 1% genome length apart.

Supplementary file5 (PDF 4842 KB) Supplementary Figure S5. Alignment of the Forkhead domain of all sequences from arthropods, onychophorans and tardigrades used in the analysis. On the left are the species names and accession numbers. The dashes in the alignment represent gaps introduced to improve the alignment. The colors denote the individual amino acids.

Supplementary file6 (XLSX 12 KB) Supplementary Table S1. Sources for sequence data of all species used in the phylogenetic analyses. For each species are given the access details for the sequence data: the second column gives the BioProject number and the third column gives the original source of the sequence data.

Supplementary file7 (XLSX 134 KB) Supplementary Table S2. Overview of all sequences used in the phylogenetic analyses. Column A gives the species name, column B gives the accession information for the sequence data. Column C gives the similarity of the forkhead (FHD) domain to the sequence data in the PfamA database ("FHD score"). Column D gives the accession details of the scaffold of the genomic data that includes the gene, column E and column F delimit the exact location of the gene sequence within the scaffold. Column G gives the distance to the nearest Fox gene in bp (if there is one on the same scaffold).

Supplementary file8 (XLSX 38 KB) Supplementary Table S3. Results of Fox gene sub-family assignments of panarthropod sequences. Column A gives the species name, column B gives the accession information for the sequence data. Columns C, D and E give the Fox gene sub-family the gene has been assigned to in the phylogenetic analyses: in the analysis with all opisthokont species, but only the forkhead domain (column C), the analysis of the panarthropod taxon sub-set and only the forkhead domain (column D), and the analysis of the the panarthropod taxon sub-set and the entire protein sequence (column E). Column F gives the consensus classification of the gene into a Fox gene sub-family, i.e. if the gene is placed in the same sub-family in at least 2 of the 3 analyses, then this sub-family is defined as the consensus, otherwise the gene is classified in the "orphan" category (indicated by questionmarks).

## Data Availability

All data generated or analyzed during this study are included in this published article and its supplementary information files.
