# Supplementary figures and images for: Phylogenetic analysis of forkhead transcription factors in the Panarthropoda
Source: Dev Genes Evol. 2022 Mar 1;232(1):39–48. doi: 10.1007/s00427-022-00686-3 (PMC8918179; doi:10.1007/s00427-022-00686-3)

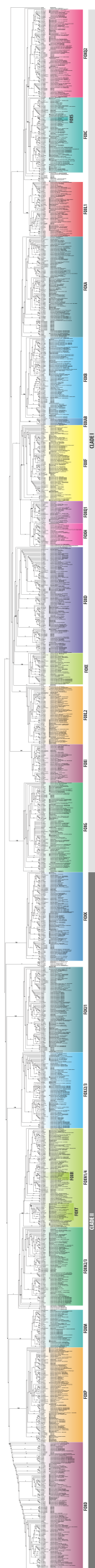

Supplement: Supplementary file 1 — Supplementary file1 (PDF 35.6 MB) Supplementary Figure S1. Unrooted phylogenetic cladogram of all Fox genes from all selected opisthokont species, based on the sequence of the forkhead domain. The colors denote the Fox-gene sub-families. "Clade I" and "Clade II" indicate the principal subdivision of Fox genes into a group with intronless forkhead domain (Clade I) and a group with the forkhead domain interrupted by at least one intron (Clade II). Species and sequence accession numbers are indicated at the terminals. Numbers at the tree edges indicate summarized bootstrap values according to the Majority Rule. The stars at the tip of the lineages denote sequences derived from arthropods, onychophorans and tardigrades. [file 427_2022_686_MOESM1_ESM.pdf]

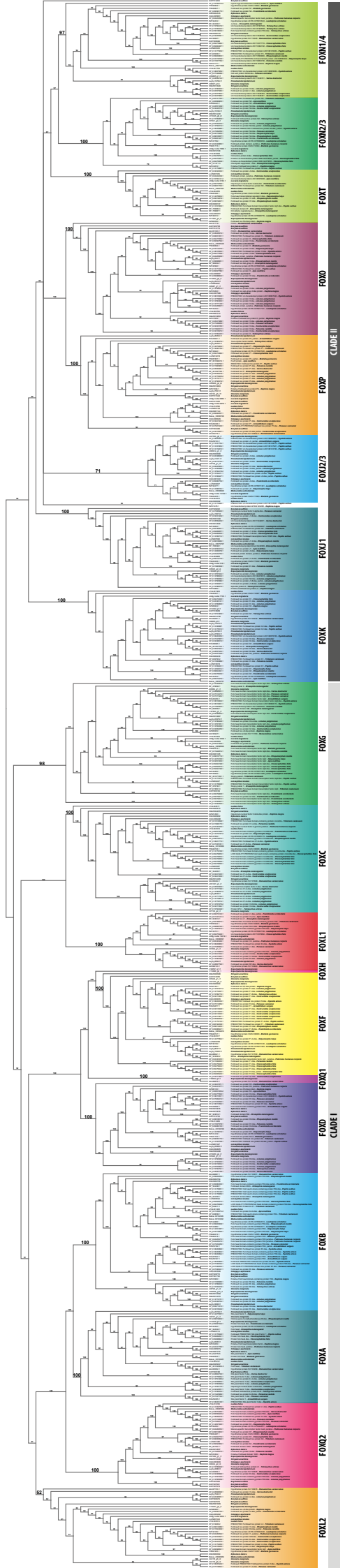

Supplement: Supplementary file 2 — Supplementary file2 (PDF 898 KB) Supplementary Figure S2. Unrooted phylogenetic cladogram of all Fox genes from representatives of the Panarthropoda, based on the sequence of the forkhead domain. The colors denote the Fox-gene sub-families. "Clade I" and "Clade II" indicate the principal subdivision of Fox genes into a group with intronless forkhead domain (Clade I) and a group with the forkhead domain interrupted by at least one intron (Clade II). Species and sequence accession numbers are indicated at the terminals. Numbers at the tree edges indicate summarized bootstrap values according to the Majority Rule. [file 427_2022_686_MOESM2_ESM.pdf]

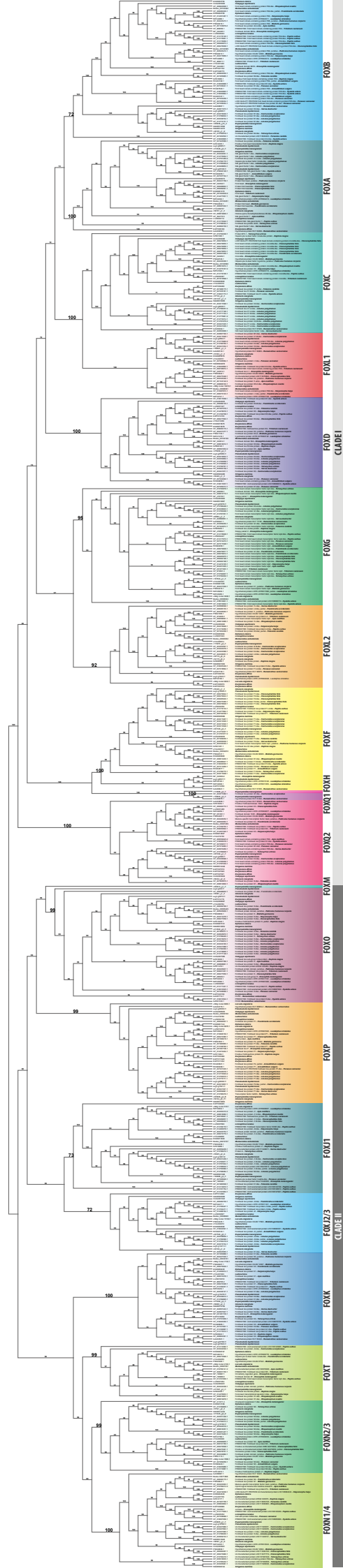

Supplement: Supplementary file 3 — Supplementary file3 (PDF 906 KB) Supplementary Figure S3. Unrooted phylogenetic cladogram of all Fox genes from representatives of the Panarthropoda, based on the entire sequence of the conceptually translated proteins. The colors denote the Fox-gene sub-families. "Clade I" and "Clade II" indicate the principal subdivision of Fox genes into a group with intronless forkhead domain (Clade I) and a group with the forkhead domain interrupted by at least one intron (Clade II). Species and sequence accession numbers are indicated at the terminals. Numbers at the tree edges indicate summarized bootstrap values according to the Majority Rule. [file 427_2022_686_MOESM3_ESM.pdf]

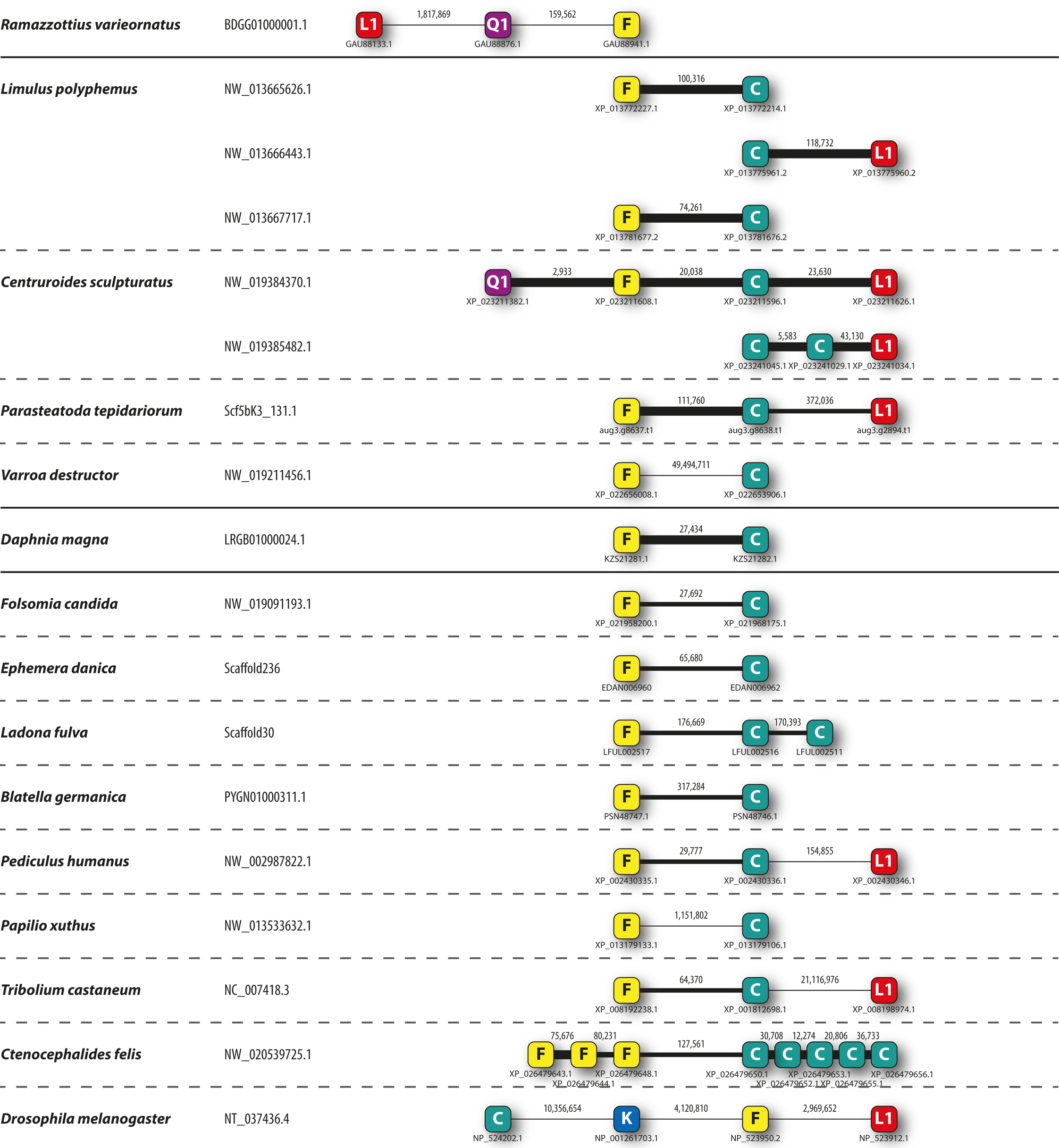

Supplement: Supplementary file 4 — Supplementary file4 (JPG 1151 KB) Supplementary Figure S4. Summary of the FoxQ1-FoxF-FoxC-FoxL1 cluster identified in the available genome sequence of diverse panarthropods. Dashed or full lines separate species. Genome sequence accessions (or chromosomes) are given in the second column, whereas accession numbers for single genes are given below the colored boxes for each gene separately. The numbers above the lines between colored boxes give the distance between the genes (in base pairs). The width of the lines indicates the relative distance: thick lines, less than 0.1% genome length apart; medium lines, less than 1% genome length apart; thin lines, over 1% genome length apart. [file 427_2022_686_MOESM4_ESM.jpg]
